# *N*-(2-Chloro­pyrimidin-4-yl)-*N*,2-di­methyl-2*H*-indazol-6-amine

**DOI:** 10.1107/S1600536810042753

**Published:** 2010-10-30

**Authors:** Hao-Fei Qi, Bing-Ni Liu, Mo Liu, Deng-Ke Liu

**Affiliations:** a Materials Science and Engineering, Tianjin Polytechnic University, Tianjin 300160, People’s Republic of China; b Tianjin Institute of Pharmaceutical Research, Tianjin 300193, People’s Republic of China

## Abstract

In the title compound, C_13_H_12_ClN_5_, which is a derivative of the anti­tumor agent pazopanib {systematic name: 5-[[4-[(2,3-di­methyl-2*H*-indazol-6-yl)methylamino]-2-pyrimidinyl]amino]-2-methylbenzolsulfonamide}, the indazole and pyrim­idine fragments form a dihedral angle of 62.63 (5)°. In the crystal, pairs of mol­ecules related by twofold rotational symmetry are linked into dimers through π–π inter­actions between the indazole ring systems [centroid–centroid distance = 3.720 (2) Å]. Weak inter­molecular C—H⋯N hydrogen bonds further assemble these dimers into columns propagated in [001].

## Related literature

For background to the pharmacokinetics and clinical studies of the anti­tumor agent pazopanib, see: Limvorasak & Posadas (2009 ▶); Sloan & Scheinfeld 2008 ▶; Sonpavde *et al.* (2007 ▶). For the synthesis of pazopanib, see: Sorbera *et al.* (2006 ▶).
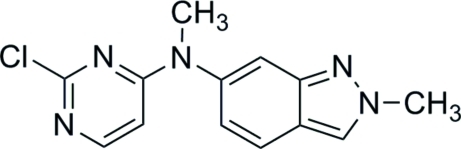


## Experimental

### 

#### Crystal data

C_13_H_12_ClN_5_
*M* *_r_* = 273.73
Monoclinic, 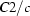

*a* = 21.432 (4) Å
*b* = 9.836 (2) Å
*c* = 12.542 (3) Å
β = 90.25 (3)°
*V* = 2644.1 (9) Å^3^
*Z* = 8
Mo *K*α radiation
μ = 0.28 mm^−1^
*T* = 113 K
0.20 × 0.18 × 0.12 mm

#### Data collection

Rigaku Saturn CCD area-detector diffractometer
Absorption correction: multi-scan (*CrystalClear*; Rigaku/MSC, 2005 ▶) *T*_min_ = 0.946, *T*_max_ = 0.967
10576 measured reflections
2323 independent reflections
1982 reflections with *I* > 2σ(*I*)
*R*_int_ = 0.043

#### Refinement

*R*[*F* ^2^ > 2σ(*F* ^2^)] = 0.036
*wR*(*F* ^2^) = 0.100
*S* = 1.01
2323 reflections
175 parameters
H-atom parameters constrained
Δρ_max_ = 0.21 e Å^−3^
Δρ_min_ = −0.25 e Å^−3^

### 

Data collection: *CrystalClear* (Rigaku/MSC, 2005 ▶); cell refinement: *CrystalClear*; data reduction: *CrystalClear*; program(s) used to solve structure: *SHELXS97* (Sheldrick, 2008 ▶); program(s) used to refine structure: *SHELXL97* (Sheldrick, 2008 ▶); molecular graphics: *SHELXTL* (Sheldrick, 2008 ▶); software used to prepare material for publication: *SHELXTL*.

## Supplementary Material

Crystal structure: contains datablocks global, I. DOI: 10.1107/S1600536810042753/cv2775sup1.cif

Structure factors: contains datablocks I. DOI: 10.1107/S1600536810042753/cv2775Isup2.hkl

Additional supplementary materials:  crystallographic information; 3D view; checkCIF report

## Figures and Tables

**Table 1:** Hydrogen-bond geometry (Å, °)

*D*—H⋯*A*	*D*—H	H⋯*A*	*D*⋯*A*	*D*—H⋯*A*
C13—H13*B*⋯N2^i^	0.98	2.56	3.517 (2)	166

Symmetry code: (i) 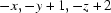.

**Fig. 1.**
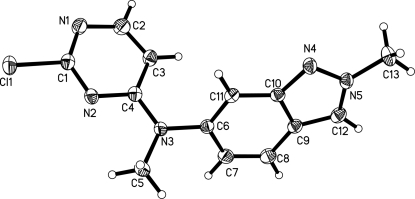
The molecular structure of (I). Displacement ellipsoids are drawn at the 50% probability level.

## supplementary crystallographic information

### Comment

Pazopanib is an oral, second-generation multi-targeted tyrosine kinase inhibitor that targets VEGFR, platelet-derived growth factor receptor and c-kit, key proteins responsible for tumor growth and survival (Limvorasak *et al.*, 2009; Sloan *et al.*, 2008; Sonpavde *et al.*, 2007). The crystal structure of the title compound (I), a derivative of pazopanib, synthesized through the transformation of pazopanib (Sorbera *et al.*, 2006), is reported here.

In (I) (Fig. 1), the indazole and pyrimidine fragments form a dihedral angle of 62.63 (5)°. In the crystal structure, The π–π contacts between the indazole systems from the adjacent molecules (Table 1) link them into dimers. Weak intermolecular C—H···N hydrogen bonds (Table 2) link further the dimers into columns propagated in direction [001].

### Experimental

To a stirred solution of the *N*-(2-chloropyrimidin-4-yl)-2 -methyl-2*H*-indazol-6-amine 5 g (0.02 mol) in DMF (30 ml) was added Cs_2_CO_3_ 9.8 g (0.03 mol) and iodomethane 2.5 ml (5.7 g, 0.04 mol) at room temperature. The mixture was stirred for 5 h. The reaction mixture was then poured into an ice-water bath, and the precipitate was collected *via* filtration and washed with water. The precipitate was air-dried to get off-white solid as crude product. The solid was dissolved in ethyl acetate 30 ml at 278 k, then white crystals were generated slowly.

### Refinement

C-bound H atoms were geometrically positioned (C—H 0.95–0.98 Å), and refined as riding with *U*_iso_ = 1.2-1.5 *U*_eq_(C).

### Crystal data

**Table:**

C_13_H_12_ClN_5_	*F*(000) = 1136
*M**_r_* = 273.73	*D*_x_ = 1.375 Mg m^−^^3^
Monoclinic, *C*2/*c*	Mo *K*α radiation, λ = 0.71073 Å
*a* = 21.432 (4) Å	Cell parameters from 4286 reflections
*b* = 9.836 (2) Å	θ = 1.9–27.9°
*c* = 12.542 (3) Å	µ = 0.28 mm^−^^1^
β = 90.25 (3)°	*T* = 113 K
*V* = 2644.1 (9) Å^3^	Block, white
*Z* = 8	0.20 × 0.18 × 0.12 mm

### Data collection

**Table:**

Rigaku Saturn CCD area-detector diffractometer	2323 independent reflections
Radiation source: rotating anode	1982 reflections with *I* > 2σ(*I*)
confocal	*R*_int_ = 0.043
Detector resolution: 7.31 pixels mm^-1^	θ_max_ = 25.0°, θ_min_ = 1.9°
ω and φ scans	*h* = −25→25
Absorption correction: multi-scan (*CrystalClear*; Rigaku/MSC, 2005)	*k* = −11→11
*T*_min_ = 0.946, *T*_max_ = 0.967	*l* = −14→14
10576 measured reflections	

### Refinement

**Table:**

Refinement on *F*^2^	Secondary atom site location: difference Fourier map
Least-squares matrix: full	Hydrogen site location: inferred from neighbouring sites
*R*[*F*^2^ > 2σ(*F*^2^)] = 0.036	H-atom parameters constrained
*wR*(*F*^2^) = 0.100	*w* = 1/[σ^2^(*F*_o_^2^) + (0.070*P*)^2^] where *P* = (*F*_o_^2^ + 2*F*_c_^2^)/3
*S* = 1.01	(Δ/σ)_max_ = 0.001
2323 reflections	Δρ_max_ = 0.21 e Å^−^^3^
175 parameters	Δρ_min_ = −0.25 e Å^−^^3^
0 restraints	Extinction correction: *SHELXL97* (Sheldrick, 2008), Fc^*^=kFc[1+0.001xFc^2^λ^3^/sin(2θ)]^-1/4^
Primary atom site location: structure-invariant direct methods	Extinction coefficient: 0.0191 (14)

### Special details

**Table:**

Geometry. All e.s.d.'s (except the e.s.d. in the dihedral angle between two l.s. planes) are estimated using the full covariance matrix. The cell e.s.d.'s are taken into account individually in the estimation of e.s.d.'s in distances, angles and torsion angles; correlations between e.s.d.'s in cell parameters are only used when they are defined by crystal symmetry. An approximate (isotropic) treatment of cell e.s.d.'s is used for estimating e.s.d.'s involving l.s. planes.
Refinement. Refinement of *F*^2^ against ALL reflections. The weighted *R*-factor *wR* and goodness of fit *S* are based on *F*^2^, conventional *R*-factors *R* are based on *F*, with *F* set to zero for negative *F*^2^. The threshold expression of *F*^2^ > σ(*F*^2^) is used only for calculating *R*-factors(gt) *etc*. and is not relevant to the choice of reflections for refinement. *R*-factors based on *F*^2^ are statistically about twice as large as those based on *F*, and *R*-factors based on ALL data will be even larger.

### Fractional atomic coordinates and isotropic or equivalent isotropic displacement parameters (Å^2^)

**Table:**

	*x*	*y*	*z*	*U*_iso_*/*U*_eq_	
Cl1	0.227613 (19)	0.06867 (4)	0.79285 (3)	0.0308 (2)	
N1	0.13815 (6)	0.18363 (13)	0.68576 (10)	0.0268 (4)	
N2	0.18239 (6)	0.30697 (12)	0.83256 (10)	0.0206 (3)	
N3	0.14793 (6)	0.52060 (13)	0.88097 (10)	0.0206 (3)	
N4	−0.06548 (6)	0.69175 (13)	0.92048 (10)	0.0223 (3)	
N5	−0.08472 (6)	0.82333 (13)	0.91397 (10)	0.0218 (3)	
C1	0.17633 (7)	0.20335 (15)	0.76669 (12)	0.0211 (4)	
C2	0.10043 (8)	0.29266 (17)	0.66821 (13)	0.0284 (4)	
H2	0.0720	0.2880	0.6101	0.034*	
C3	0.10066 (8)	0.40761 (16)	0.72803 (12)	0.0235 (4)	
H3	0.0736	0.4815	0.7124	0.028*	
C4	0.14298 (7)	0.41252 (15)	0.81461 (12)	0.0189 (4)	
C5	0.19380 (7)	0.52028 (18)	0.96815 (13)	0.0295 (4)	
H5A	0.1776	0.4671	1.0280	0.044*	
H5B	0.2016	0.6139	0.9915	0.044*	
H5C	0.2329	0.4797	0.9432	0.044*	
C6	0.10170 (7)	0.62570 (16)	0.88222 (11)	0.0194 (4)	
C7	0.12160 (7)	0.76193 (16)	0.86613 (13)	0.0252 (4)	
H7	0.1644	0.7801	0.8529	0.030*	
C8	0.07992 (7)	0.86753 (17)	0.86940 (13)	0.0267 (4)	
H8	0.0935	0.9585	0.8592	0.032*	
C9	0.01641 (7)	0.83811 (15)	0.88831 (12)	0.0209 (4)	
C10	−0.00297 (7)	0.70070 (15)	0.90426 (11)	0.0188 (4)	
C11	0.04067 (7)	0.59365 (15)	0.90219 (12)	0.0195 (4)	
H11	0.0282	0.5023	0.9142	0.023*	
C12	−0.03903 (7)	0.91238 (17)	0.89500 (12)	0.0246 (4)	
H12	−0.0434	1.0080	0.8875	0.030*	
C13	−0.15098 (7)	0.85326 (18)	0.92276 (13)	0.0286 (4)	
H13A	−0.1573	0.9519	0.9201	0.043*	
H13B	−0.1667	0.8179	0.9906	0.043*	
H13C	−0.1735	0.8102	0.8636	0.043*	

### Atomic displacement parameters (Å^2^)

**Table:**

	*U*^11^	*U*^22^	*U*^33^	*U*^12^	*U*^13^	*U*^23^
Cl1	0.0287 (3)	0.0271 (3)	0.0365 (3)	0.00976 (17)	−0.00266 (19)	−0.00407 (17)
N1	0.0329 (8)	0.0226 (8)	0.0250 (8)	0.0042 (6)	−0.0049 (6)	−0.0020 (6)
N2	0.0177 (7)	0.0222 (8)	0.0220 (7)	0.0014 (6)	0.0025 (5)	0.0001 (6)
N3	0.0176 (7)	0.0228 (7)	0.0214 (7)	0.0028 (6)	−0.0009 (5)	−0.0045 (6)
N4	0.0207 (7)	0.0194 (8)	0.0267 (8)	0.0035 (6)	0.0006 (6)	0.0018 (5)
N5	0.0223 (7)	0.0204 (7)	0.0226 (7)	0.0049 (6)	−0.0011 (5)	0.0004 (6)
C1	0.0205 (8)	0.0200 (9)	0.0228 (9)	0.0009 (7)	0.0050 (7)	0.0024 (7)
C2	0.0344 (9)	0.0284 (10)	0.0225 (9)	0.0018 (8)	−0.0080 (7)	0.0005 (7)
C3	0.0274 (9)	0.0220 (9)	0.0210 (8)	0.0041 (7)	−0.0025 (7)	0.0036 (7)
C4	0.0181 (8)	0.0203 (9)	0.0184 (8)	−0.0010 (6)	0.0052 (6)	0.0028 (6)
C5	0.0229 (9)	0.0349 (10)	0.0307 (10)	0.0057 (8)	−0.0078 (7)	−0.0107 (8)
C6	0.0206 (8)	0.0214 (9)	0.0163 (8)	0.0019 (7)	−0.0011 (6)	−0.0024 (6)
C7	0.0212 (8)	0.0254 (9)	0.0290 (9)	−0.0040 (7)	0.0022 (7)	−0.0015 (7)
C8	0.0278 (9)	0.0194 (9)	0.0328 (10)	−0.0036 (7)	0.0007 (7)	0.0011 (7)
C9	0.0239 (8)	0.0188 (8)	0.0200 (8)	−0.0002 (7)	−0.0013 (6)	0.0001 (6)
C10	0.0201 (8)	0.0199 (8)	0.0164 (8)	−0.0004 (6)	−0.0025 (6)	−0.0002 (6)
C11	0.0225 (8)	0.0176 (8)	0.0185 (8)	−0.0005 (6)	−0.0001 (6)	−0.0003 (6)
C12	0.0303 (10)	0.0180 (8)	0.0255 (9)	0.0009 (7)	−0.0013 (7)	0.0003 (7)
C13	0.0223 (9)	0.0308 (10)	0.0328 (10)	0.0082 (7)	0.0023 (7)	0.0053 (7)

### Geometric parameters (Å, °)

**Table:**

Cl1—C1	1.7515 (16)	C5—H5B	0.9800
N1—C1	1.315 (2)	C5—H5C	0.9800
N1—C2	1.360 (2)	C6—C11	1.370 (2)
N2—C1	1.3182 (19)	C6—C7	1.421 (2)
N2—C4	1.3564 (19)	C7—C8	1.371 (2)
N3—C4	1.3540 (19)	C7—H7	0.9500
N3—C6	1.4321 (19)	C8—C9	1.413 (2)
N3—C5	1.467 (2)	C8—H8	0.9500
N4—C10	1.3586 (19)	C9—C12	1.398 (2)
N4—N5	1.3607 (17)	C9—C10	1.428 (2)
N5—C12	1.336 (2)	C10—C11	1.409 (2)
N5—C13	1.4551 (19)	C11—H11	0.9500
C2—C3	1.357 (2)	C12—H12	0.9500
C2—H2	0.9500	C13—H13A	0.9800
C3—C4	1.413 (2)	C13—H13B	0.9800
C3—H3	0.9500	C13—H13C	0.9800
C5—H5A	0.9800		
			
Cg1···Cg2^i^	3.720 (2)		
			
C1—N1—C2	112.08 (13)	C11—C6—C7	122.04 (14)
C1—N2—C4	115.36 (12)	C11—C6—N3	119.81 (14)
C4—N3—C6	121.42 (12)	C7—C6—N3	118.11 (13)
C4—N3—C5	120.45 (13)	C8—C7—C6	120.96 (15)
C6—N3—C5	117.04 (12)	C8—C7—H7	119.5
C10—N4—N5	103.19 (12)	C6—C7—H7	119.5
C12—N5—N4	114.34 (13)	C7—C8—C9	118.58 (15)
C12—N5—C13	126.67 (14)	C7—C8—H8	120.7
N4—N5—C13	118.92 (13)	C9—C8—H8	120.7
N1—C1—N2	131.07 (14)	C12—C9—C8	136.31 (15)
N1—C1—Cl1	114.88 (12)	C12—C9—C10	103.78 (14)
N2—C1—Cl1	114.05 (11)	C8—C9—C10	119.90 (14)
C3—C2—N1	124.52 (14)	N4—C10—C11	127.55 (14)
C3—C2—H2	117.7	N4—C10—C9	111.71 (13)
N1—C2—H2	117.7	C11—C10—C9	120.74 (14)
C2—C3—C4	117.01 (15)	C6—C11—C10	117.78 (14)
C2—C3—H3	121.5	C6—C11—H11	121.1
C4—C3—H3	121.5	C10—C11—H11	121.1
N3—C4—N2	116.87 (13)	N5—C12—C9	106.98 (14)
N3—C4—C3	123.20 (14)	N5—C12—H12	126.5
N2—C4—C3	119.91 (14)	C9—C12—H12	126.5
N3—C5—H5A	109.5	N5—C13—H13A	109.5
N3—C5—H5B	109.5	N5—C13—H13B	109.5
H5A—C5—H5B	109.5	H13A—C13—H13B	109.5
N3—C5—H5C	109.5	N5—C13—H13C	109.5
H5A—C5—H5C	109.5	H13A—C13—H13C	109.5
H5B—C5—H5C	109.5	H13B—C13—H13C	109.5
			
C10—N4—N5—C12	0.03 (17)	C11—C6—C7—C8	−0.4 (2)
C10—N4—N5—C13	177.21 (12)	N3—C6—C7—C8	−178.15 (13)
C2—N1—C1—N2	1.9 (2)	C6—C7—C8—C9	−0.5 (2)
C2—N1—C1—Cl1	−178.80 (12)	C7—C8—C9—C12	−178.16 (16)
C4—N2—C1—N1	−0.3 (2)	C7—C8—C9—C10	0.4 (2)
C4—N2—C1—Cl1	−179.62 (10)	N5—N4—C10—C11	−179.72 (13)
C1—N1—C2—C3	−1.4 (2)	N5—N4—C10—C9	0.32 (16)
N1—C2—C3—C4	−0.4 (3)	C12—C9—C10—N4	−0.53 (17)
C6—N3—C4—N2	−168.27 (13)	C8—C9—C10—N4	−179.54 (12)
C5—N3—C4—N2	−0.5 (2)	C12—C9—C10—C11	179.50 (13)
C6—N3—C4—C3	13.5 (2)	C8—C9—C10—C11	0.5 (2)
C5—N3—C4—C3	−178.74 (15)	C7—C6—C11—C10	1.3 (2)
C1—N2—C4—N3	179.92 (13)	N3—C6—C11—C10	179.02 (12)
C1—N2—C4—C3	−1.8 (2)	N4—C10—C11—C6	178.69 (14)
C2—C3—C4—N3	−179.73 (15)	C9—C10—C11—C6	−1.3 (2)
C2—C3—C4—N2	2.1 (2)	N4—N5—C12—C9	−0.37 (18)
C4—N3—C6—C11	57.33 (19)	C13—N5—C12—C9	−177.29 (13)
C5—N3—C6—C11	−110.80 (17)	C8—C9—C12—N5	179.28 (16)
C4—N3—C6—C7	−124.86 (16)	C10—C9—C12—N5	0.52 (16)
C5—N3—C6—C7	67.01 (18)		

Symmetry codes: (i) −*x*, *y*, −*z*+3/2.

### Hydrogen-bond geometry (Å, °)

**Table:**

*D*—H···*A*	*D*—H	H···*A*	*D*···*A*	*D*—H···*A*
C13—H13B···N2^ii^	0.98	2.56	3.517 (2)	166

Symmetry codes: (ii) −*x*, −*y*+1, −*z*+2.
